# Large interfacial contribution to ultrafast THz emission by inverse spin Hall effect in CoFeB/Ta heterostructure

**DOI:** 10.1016/j.isci.2022.104718

**Published:** 2022-07-04

**Authors:** Sandeep Kumar, Sunil Kumar

**Affiliations:** 1 Femtosecond Spectroscopy and Nonlinear Photonics Laboratory, Department of Physics, Indian Institute of Technology Delhi, New Delhi 110016, India

**Keywords:** Physics, Hall effect, Radiation physics

## Abstract

Ultrafast THz radiation generation from ferromagnetic/nonmagnetic (FM/NM) spintronic heterostructures generally exploits the spin-charge conversion within the nonmagnetic layer and its interface with the ferromagnetic layer. Various possible sub-contributions to the underlying mechanism need to be exploited not only for investigating the intricacies at the fundamental level in the material properties themselves but also for improving their performance for broadband and high-power THz emission. Here, we report ultrafast THz emission from (CoFeB,Fe)/(Ta,Pt) bilayers at varying sample temperatures to unravel the role of intrinsic and extrinsic spin-charge conversion processes through the extracted values of spin-Hall conductivities. An enhanced THz emission along with temperature-dependent THz signal polarity reversal is observed in the case of annealed CoFeB/Ta. These results demonstrate a large interfacial contribution to the overall spin-Hall angle arising from the modified interface in the annealed CoFeB/Ta.

## Highlights

• Temperature-dependent THz emission from spintronic heterostructures
• THz polarity reversal at a certain temperature in the annealed CoFeB/Ta
• Determination of spin-Hall conductivity from the efficiency of THz emission
• Distinguishing the intrinsic/extrinsic contributions to spin-charge conversion

## Graphical abstract

**Figure:**
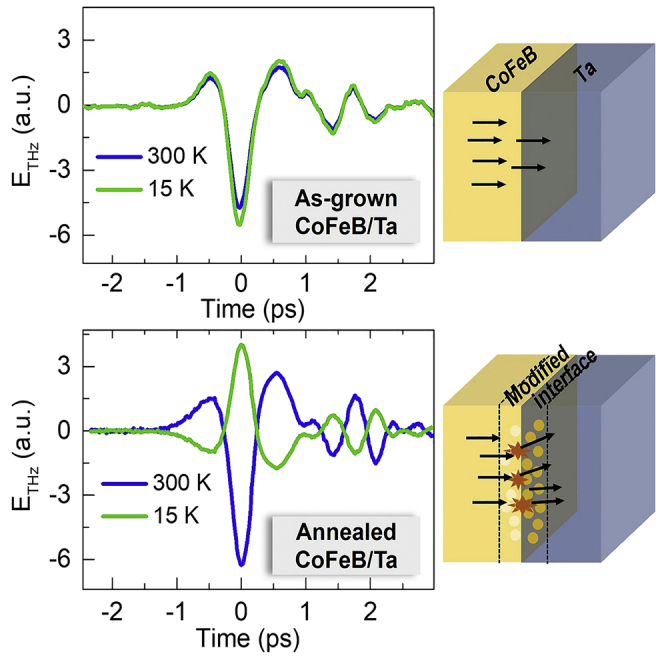

## Introduction

Spin-orbit coupling (SOC) (Dyakonov and Perel, 1971) in solids is the origin of various interesting relativistic phenomena, such as the spin Hall effect (SHE) and its inverse (ISHE) (Sinova et al., 2015; Hoffmann, 2013), the spin-orbit torque (Garello et al., 2013) (SOT), Rashba-Edelstein effect (REE) and its inverse (IREE) (Edelstein, 1990; Bychkov and Rashba É, 1984). Spin-Hall angle is the measurable quantity that determines the charge to spin or spin-to-charge conversion in the above processes. In spintronic devices, where SHE and its inverse (ISHE) are the leading contributors, the generation, manipulation, and detection of the spin current can be effectively conducted by electrical (Hirsch, 1999; Niimi and Otani, 2015) and optical means (van ‘t Erve et al., 2014; Kampfrath et al., 2013). It is always desirous to obtain spintronic materials, their appropriate combinations, and heterostructures, which can provide a large spin (charge) to charge (spin) current conversion efficiency so as to implement them in practical applications. For designing such spintronic material structures with large spin Hall angle, it is necessary to understand the underlying fundamental processes. In the SHE, both the extrinsic and the intrinsic mechanisms play a pivotal role and a distinction between the two can be decisive for specific applications. The intrinsic processes include spin Berry curvature relating to the deflection of spins in the electronic band structure of perfectly ordered materials (Karplus and Luttinger, 1954). The extrinsic term is related to the disorder-induced scattering events owing to the localized impurities. The corresponding contribution to the overall SHE is recognized as skew scattering (Smit, 1958) and side-jump scattering (Berger, 1970). Disentanglement of each contribution in the total SHE is important so as to enhance the spin-charge (or charge-spin) conversion efficiency and hence the ultrafast terahertz (THz) radiation generation from ferromagnetic/nonmagnetic (FM/NM) bilayer heterostructure-based spintronic THz emitters, where the NM layer is typically a heavy metal. Temperature-dependent experiments for physical property measurements are conducted routinely for this purpose on heavy metals (Sagasta et al., 2016, 2018; Wang et al., 2016; Hao and Xiao, 2015). By determining the temperature dependence of spin Hall resistivity behavior (and/or conductivity) and spin Hall angle, the contribution of the dominant mechanism can be quantified. For instance, from spin absorption experiments providing a value of the intrinsic spin Hall conductivity to be −820 ± 120 (*ħ/e*) Ω^−1^cm^−1^ in high-resistive β-phase tantalum (Ta), the intrinsic mechanism is dominant (Sagasta et al., 2018). Similarly, from resistivity measurements on another popular NM heavy metal, platinum (Pt), the dominance of the intrinsic mechanism over the extrinsic one and vice-versa, depending on the spin Hall conductivity, has been determined (Sagasta et al., 2016). In multilayer structures, the interface and its quality can also affect the extrinsic contributions drastically. It has been seen that the extrinsic contribution to SHE and ISHE is many times higher in Py/Pt than that in bulk Pt (Nguyen et al., 2016; Hou et al., 2012).

For probing the spin-charge conversion efficiency and the corresponding underlying mechanisms, there exist well-established transport methods (Cramer et al., 2018; Avci et al., 2014a; Sagasta et al., 2018; Nguyen et al., 2016), such as the harmonic Hall measurements, ferromagnetic resonance (FMR)-based techniques, spin absorption technique, and DC spin Seebeck effect. In recent years, THz emission time-domain spectroscopy has become another more promising technique for these measurements in a non-destructive and contactless manner (Cheng et al., 2019, 2021; Matthiesen et al., 2020). Either the spin-to-charge conversion in spintronic structures is directly seen in terms of the THz emission efficiency or THz pulses can be employed in time-domain spectroscopy to characterize them (Agarwal et al., 2021). For example, temperature-dependent THz emission studies were used to determine the dominant intrinsic contribution to the spin-charge conversion mechanism in Co/Pt bilayer heterostructure (Matthiesen et al., 2020). Similarly, it has also been utilized as a probe to identify the dominance of skew scattering at the interface of various spintronic heterostructures (Gueckstock et al., 2021). Most recently, laser-induced THz emission spectroscopy has been used as a fingerprint identification experimental scheme to disentangle the ISHE from IREE in Ag/Biheterostructure (Shen et al., 2021), which had otherwise been known before as the Rashba interface material heterostructure having only IREE (Jungfleisch et al., 2018; Zhang et al., 2015). Ta is a popular choice as the NM layer in many FM/NM-based spintronic devices. Even more, Ta is routinely used as a capping or buffer layer. It can be grown in either α− or β− or mixed phase, where the spin Hall angle and the resistivity show a dramatic variation from one phase to the other (Kumar et al., 2018). A large spin Hall angle value makes this NM heavy metal the popular choice in varieties of SHE and ISHE-based spintronic devices. In CoFeB/Ta bilayer heterostructure, a large magnetization reversal (Liu et al., 2012) and tunnel magnetoresistance (Ikeda et al., 2010) have been realized to make this FM/NM combination an appropriate choice in the field of spintronics and also a significant one for the generation of THz radiation by ultrafast optical excitation. Moreover, the annealing of such structure strongly modifies the material and interfacial properties (Sasaki et al., 2017, 2019) which ultimately affect spin-charge mechanism-related parameters. In addition, CoFeB also owns properties (Seifert et al., 2016; Cheng et al., 2022) like good spin injection, and very weak sensitivity toward temperature variation for resistivity, magnetization, and self-SHE, and so forth. Its heterostructure with Ta, an NM having low bulk SOC strength, can therefore help observe substantial interfacial spin-to-charge conversion (Hawecker et al., 2021) and its temperature-dependent behavior.

In this article, we report temperature-dependent THz emission from thickness optimized CoFeB/Ta and Fe/Pt bilayer spintronic heterostructures, which help us to quantitatively distinguish between distinct contributions to the spin-charge conversion mechanism in CoFeB/Ta spintronic THz emitter. By combining the temperature-dependent resistivity and THz emission measurements, we show that in the as-grown bilayer samples, i.e., CoFeB/Ta and Fe/Pt, the ISHE is driven by the dominating intrinsic contribution. On the other hand, for the annealed CoFeB/Ta sample, a sign reversal of the spin Hall conductivity is manifested from the experimentally observed polarity reversed THz emission below a certain temperature. This peculiar behavior is attributed to the dominance of the interfacial contribution over the intrinsic bulk one owing to the interfacial modification in the annealed sample.

## Results

**Figure 1:**
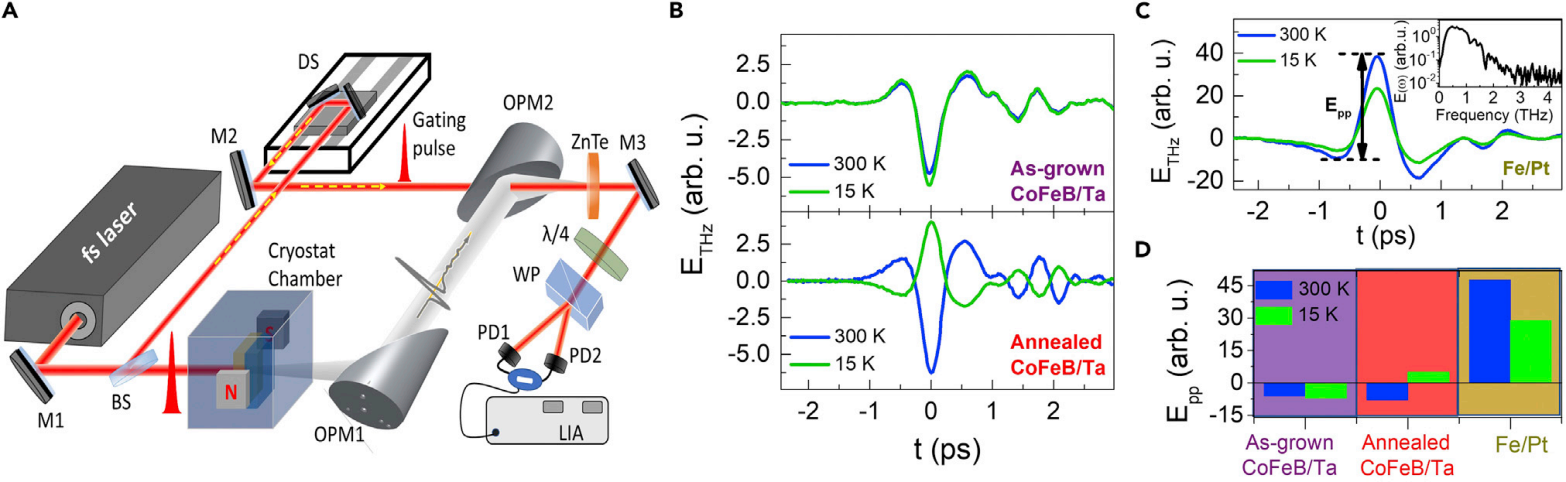
THz pulse generation from femtosecond NIR (800 nm) pulse irradiated FM/NM bilayer-type spintronic emitter deposited on HR-Si substrates. BS: beam splitter, M: mirror, DS: delay stage, OPMs: off-axis parabolic mirrors, λ/4: quarter-wave plate, NC: nonlinear optical crystal, WP: Wollaston prism, BPD: balanced photodiode, LIA: lock-in amplifier. (A) Schematic of the cryogenically combined experimental setup for temperature-dependent THz time-domain spectroscopic measurements. (B) Comparison between THz time-domain signals from the as-grown CoFeB/Ta, annealed CoFeB/Ta, and Fe/Pt bilayer spintronic heterostructures at temperature values of 15 and 300 K. The inset shows the corresponding Fourier spectra. (C) Peak-to-peak THz signal amplitude (E_pp_) of the as-grown CoFeB/Ta, annealed CoFeB/Ta, and Fe/Pt THz emitters for the temperature values 15 and 300 K.

Typical time-domain THz electro-optic signals generated from the as-grown CoFeB/Ta and the annealed CoFeB/Ta bilayer spintronic emitters are presented in Figure 1B for two extreme temperatures, i.e., 300 and 15 K. Results from experiments on Fe/Pt under the same experimental conditions of optical excitation, detection, and these two sample temperatures, are shown in Figure 1C. The spintronic heterostructures were optically excited from the NM side of the heterostructure on the substrate, i.e., the order was, light > NM/FM/substrate. The procedure for extracting the THz electric field (E_THz_) from the experimentally measured time-domain electro-optic signal is provided in the STAR methods section (see method details). Nevertheless, as the gating pulse duration (∼50 fs) in our experiments is very small, the electro-optic signal itself can be taken as E_THz_. The THz bandwidth in our experiments on both the Fe/Pt and CoFeB/Ta bilayers is nearly the same, and it is shown in the inset of Figure 1C. Various dips in the Fourier spectrum at different frequencies are owing to absorption in moisture, as all the measurements reported in this article have been performed under normal room humidity conditions (Xin et al., 2006). For a comparison between the THz generation efficiency of Fe/Pt and CoFeB/Ta, the peak-to-peak amplitude (E_pp_) of the respective E_THz_(t) signals as presented in Figures 1B and 1C, respectively, are plotted in the bar plot in Figure 1D. For all of our discussion in the following, the E_PP_ has been defined as shown in Figure 1C, where –E_PP_ means polarity reversed THz signal and E_PP_ = 0 means no THz signal. The THz pulse shape and width were nearly unchanged for all the samples measured in our experiments. Hence, E_pp_ provides an unambiguous measure of the THz amplitude for each case. The detailed temperature-dependent behavior of the THz emission from Fe/Pt and CoFeB/Ta is discussed later in the article; however, the major points to highlight from Figures 1B–1D are: (i) polarity of the THz signal from annealed CoFeB/Ta (-E_PP_) is reversed to that from the as-grown CoFeB/Ta (+E_PP_), (ii) the THz signal magnitude is temperature-dependent, and (iii) the THz signal magnitude from as-grown or annealed CoFeB/Ta is nearly 1/10^th^ of that from the Fe/Pt at both the extreme temperatures of 300 and 15 K. From Figure 1C, we also note that at the room temperature (300 K) the THz signal from the annealed CoFeB/Ta is nearly 1.3 times stronger than that from the as-grown CoFeB/Ta. This particular observation at room temperature is in close consistency with another study from the recent literature (Sasaki et al., 2017), and hence the enhancement in the THz emission from the annealed CoFeB/Ta sample can be attributed to the recrystallization followed after boron diffusion into the Ta side during annealing.

**Figure 2:**
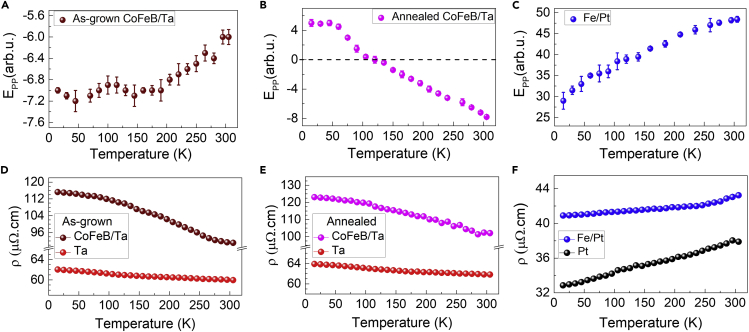
Variation of the magnitude of the THz signal (E_pp_) and the electrical resistivity (ρ) with respect to the sample temperature changing from room temperature (300 K) to very low values (A–C) E_pp_ of the as-grown CoFeB/Ta, the annealed CoFeB/Ta, and the Fe/Pt, respectively. The error bars represent the maximum experimental error in the measure THz signal. (D–F) ρ of the as-grown CoFeB/Ta and Ta, the annealed CoFeB/Ta and Ta, and Fe/Pt and Pt, respectively.

Figure 2 illustrates the temperature-dependence of the E_PP_(THz) and resistivity (ρ) of the as-grown CoFeB/Ta, annealed CoFeB/Ta, and Fe/Pt heterostructures in a wide range (15–300 K) of the sample temperature. It can be seen from Figures 2A and 2B that with the decreasing temperature, the THz amplitude for the as-grown CoFeB/Ta (-E_PP_) increases monotonically, while for the annealed CoFeB/Ta, it decreases continuously until a certain temperature only beyond which the polarity gets reversed, and the signal starts to increase again. More precisely, for the annealed CoFeB/Ta, the –E_PP_ signal at high temperatures changes to + E_PP_ signal at low temperatures. On the other hand, the case with annealed Fe/Pt is a separate question in itself and has been addressed partially in a couple of recent articles including ours (Kumar and Kumar, 2022; Scheuer et al., 2022), though the temperature-dependent measurements are yet to be conducted.

As clear from Figure 2C, the THz signal (+E_PP_) simply decreases continuously with the decreasing temperature in Fe/Pt. Such a characteristic temperature dependence in the THz emission has been previously seen Co/Pt also, another Pt-based spintronic THz emitter (Matthiesen et al., 2020). The plateau-like feature below ∼120 K as seen for the as-grown CoFeB/Ta in Figure 2A and is similar to that observed for Co/Pt earlier (Matthiesen et al., 2020), and such behaviour can arise from the choice of the FM material layer used. As HR-Si has been shown to have nearly temperature-independent properties (Lui and Hegmann, 2003) e.g., carrier density, relaxation time, and so forth, in a large range, therefore, we have ignored its impact on the temperature-dependent THz emission behavior of the spintronic emitters in the current study.

The polarization of the emitted THz radiation from spintronic emitters is basically controlled by the applied magnetic field direction ($\hat{m}$), excitation geometry to define the spin current direction (J_s_), and the spin Hall angle (θ) (Torosyan et al., 2018; Seifert et al., 2016; Kampfrath et al., 2013; Papaioannou and Beigang, 2021). All these parameters are combined in the inverse SHE mediated charge current relation, i.e., $\vec{J_{c}}=\;\theta .\;\left(\;\vec{J_{s}}\times \hat{m}\right)$. Both, the applied magnetic field and sample geometry were kept the same in our experiments for all the sample types. Therefore, the THz polarity reversal at a certain temperature, as seen in the annealed CoFeB/Ta (Figure 2B), must originate from a similar type of trend in the temperature dependence, including the sign reversal, if any, of the material’s spin Hall angle. The one-to-one correlation between the temperature-dependent behavior of THz emission from the as-grown CoFeB/Ta and the spin Hall angle of Ta in the CoFeB/Ta structure reported in the literature (Hao and Xiao, 2015), strengthens the above point. The total spin Hall angle can be expressed as a function of bulk and interfacial part of spin Hall angles, where interfacial spin Hall angle can change from negative to positive or vice-versa, with the temperature variation (Cecot et al., 2017). This has been further discussed in detail for our case later in the article.

For the NM heavy metal (NM or HM = Ta and Pt in our case), the spin Hall angle is related to the spin Hall resistivity (ρ_SH_) and the electrical resistivity (ρ_NM_) via the relation (Sagasta et al., 2016, 2018), ρ_SH_ = (θ. ρ_NM_). The temperature dependence of both the ρ_SH_ and ρ_NM_ can help in determining the behavior of θ in the entire temperature range for a given material structure (Gamou et al., 2019; Sagasta et al., 2016). Figures 2D–2F show the experimentally measured temperature dependence of the resistivity (ρ) for all the three samples under study, i.e., as-grown CoFeB/Ta, annealed CoFeB/Ta, and Fe/Pt bilayers as well as the respective NM layers (Ta and Pt) by using four-point van der Pauw method. The same could not be conducted for the CoFeB and Fe alone because of the rapid oxidation problem with these FM layers. However, it is possible to determine the resistivity of these two FM layers from the resistivity of the corresponding bilayers and the counterpart NM layers in them by using the parallel resistor model for the thin film bilayer heterostructures (Chen and Juang, 2016). According to this model, the effective resistivity of the bilayer is given by

(Equation 1) $$\rho_{T}=\left(d_{1}+d_{2}\right)\frac{\rho_{1}\rho_{2}}{d_{1}\rho_{2}+d_{2}\rho_{1}}$$

In the above, d_1,2_ and ρ_1,2_ represent the thickness and the resistivity of the individual layers in the bilayer heterostructure. Thus, an estimated mean value of the resistivity for Fe at room temperature is ∼50 μΩ cm. This value for CoFeB in the as-grown CoFeB/Ta or annealed-CoFeB/Ta is determined to be ∼162 μΩ cm, which is well-matched with the literature (Hao and Xiao, 2015).

The longitudinal resistivities of the Pt layer and Fe/Pt bilayer, as seen in Figure 2F, decrease with the decreasing temperature in a way that is typical for metallic films (Sagasta et al., 2016; Matthiesen et al., 2020). On the other hand, for Ta layer and CoFeB bilayers in Figures 2D and 2E, the resistivity increases linearly with the decreasing temperature. The negative temperature coefficient of resistance for Ta layers in Figures 2D and 2E matches well with the behavior observed in the α-phase Ta below a critical layer thickness of 4 nm (Gamou et al., 2019) and also in β-phase Ta (Hao and Xiao, 2015), and it can be related to thermally activated charge transfer processes. Among all the phases, the α-phase Ta is the low-resistive phase (Hao and Xiao, 2015; Kumar et al., 2018). The mean value of the resistivity being just ∼60 μΩ cm (Figures 2D and 2E) together with the results from the XRD measurements (see Figure S1 in the supplemental information), we confirm that the Ta layers in our case are grown in the α-phase. The resistivity of the FM layers (either Fe or CoFeB, in the present case) is nearly temperature independent throughout the temperature range considered here (Hao and Xiao, 2015). Therefore, the temperature dependence in the resistivities of the bilayers is expected to arise from that of the Pt in Fe/Pt and the Ta in CoFeB/Ta. However, a small difference in the temperature-dependent behavior of the resistivity of the bilayers as compared with the Pt or the Ta layers alone is observed in Figures 2D and 2E, and hence, it must originate from the nature of the interface (Avci et al., 2014b). In fact, we can also note a minute difference between the temperature-dependent resistivities of the as-grown CoFeB/Ta and annealed CoFeB/Ta in Figures 2D and 2E. As, the CoFeB layer alone does not have any temperature dependence (Hao and Xiao, 2015), such differences are related to the interface modifications in the systems (Sasaki et al., 2019).

Now, we discuss the results from the THz emission spectroscopy to show that the above-mentioned difference in the resistivity of the as-grown CoFeB/Ta and annealed CoFeB/Ta is, indeed, related to interface-related mechanisms. Only in a few recent studies, temperature-dependent THz time-domain spectroscopy has been employed as a probing tool to investigate the possible microscopic origin of the spin-to-charge conversion mechanism in the spintronics heterostructures (Matthiesen et al., 2020; Gueckstock et al., 2021). The amplitude of the generated THz field from the FM/NM bilayer is related to the spin current density and the spin Hall resistivity via a relation (Matthiesen et al., 2020) as $E_{THz}\left(\omega \right)=\rho_{SH}\;.\left(\frac{1}{d}.\frac{\rho_{FM/HM}}{\rho_{HM}}\right).J_{s}\left(\omega \right)$. The THz conductivity of the metallic spintronic thin films changes negligibly in a large frequency range of ∼0–4 THz (Seifert et al., 2018). Moreover, because the metallic layers with nanometer thicknesses have small parallel conductivity, the ratio $\frac{\rho_{FM/HM}}{\rho_{HM}}$ remains close to unity in the entire frequency range. Therefore, the magnitude of the emitted THz field and the spin current density can be directly compared in both the time and the frequency domains, and the equation becomes

(Equation 2) $$E_{THz}\left(t\right)=\rho_{SH}\;.\left(\frac{1}{d}.\frac{\rho_{FM/HM}}{\rho_{HM}}\right).J_{s}\left(t\right)$$

Like before, here, ρ_SH_ and ρ_NM_ represent the spin Hall resistivity and longitudinal resistivity, respectively, of the NM layer. Similarly, ρ_FM/NM_ represents the longitudinal resistivity of the FM/NM bilayer having the NM layer on the top from whose side the optical excitation takes place, *d* is the thickness of the bilayer. This relation connecting the dynamic variables (electric field and spin current) and static properties (resistivities) is valid only under the quasi-static approximation (Matthiesen et al., 2020). From Equation 2, it is evident that the THz electric field amplitude majorly depends on the spin Hall resistivity and spin current density, and hence any temperature-dependence in the magnitude of the *E*_*THz*_ can be directly related to that of the ρ_SH_ and the J_S_, provided the ratio between the electrical resistivities of the FM/NM bilayer and the NM layer, $\left(\frac{\rho_{FM/NM}}{\rho_{NM}}\right)$ remains constant. Indeed, this is true in the present case, i.e., $\left(\frac{\rho_{CoFeB/Ta}}{\rho_{Ta}}\right)$ or $\left(\frac{\rho_{Fe/Pt}}{\rho_{Pt}}\right)$ are nearly constant in the entire temperature range in Figures 2D–2F, a behavior seen even in similar other types of FM/NM bilayers in the literature (Matthiesen et al., 2020). Although the change is just a few percent, but still we have included this factor in our calculations later on. Moreover, the spin current in Equation (2) is also mostly temperature-independent because of two main reasons: (i) the magnetic phase transition temperature (Curie point) of the FM layer is much above the experimental temperatures used (Mohn and Wohlfarth, 1987), and (ii) the characteristic parameters relating to the magnetization dynamics to govern the spin current are invariant with respect to the temperature (Matthiesen et al., 2020). Therefore, the temperature-dependency in the magnitude of the THz signal can be directly attributed to the temperature-dependent variation of the spin Hall resistivity.

As pointed out earlier, not only the intrinsic but also the extrinsic mechanisms can contribute to the overall behavior of the spin Hall resistivity in a given FM/NM spintronic heterostructure. By including both the intrinsic and the extrinsic contributions, in general, the temperature-dependent spin Hall resistivity can be expressed (Sagasta et al., 2016, 2018; Tian et al., 2009) as

(Equation 3) $$\rho_{SH}\left(T\right)\;=\sigma_{HM}^{int.}.\rho_{HM}^{2}\left(T\right)+\alpha_{ss}.\rho_{0,HM}+\sigma_{SJ}.\rho_{0,HM}^{2}$$

Here, the first term on the right-hand side is the weightage of intrinsic contribution relating to the intrinsic spin Hall conductivity, $\sigma_{NM}^{int}$ and the longitudinal electrical resistivity, $\rho_{NM}$ of the NM heavy metal layer. The second and third terms represent the extrinsic contributions: the second term is for skew scattering and the third term is for side-jump scattering, both of which have been pictorially described in Figure 5. The skew scattering term is characterized by skew scattering angle, $\alpha_{ss}$ and the residual resistivity, $\rho_{0,NM}$ of the NM heavy metal layer, while, the contribution from side-jump scattering is via the corresponding side-jump spin Hall conductivity parameter, $\sigma_{SJ}$. Equations (2) and (3) can be combined for a specific time at which the THz signal is maximum, for example, to analyze the temperature-dependent contributions from the intrinsic and the extrinsic mechanisms to the generation of the THz signal for each of the FM/NM bilayers under study. The resultant can be expressed as

(Equation 4) $$\rho_{SH}\propto E_{PP}.\left(\frac{\rho_{HM}}{\rho_{FM/HM}}\right)=\sigma_{HM}^{int.}.\rho_{HM}^{2}+\sigma_{SJ}.\rho_{0,HM}^{2}+\alpha_{ss}.\rho_{0,HM}$$

**Figure 3:**
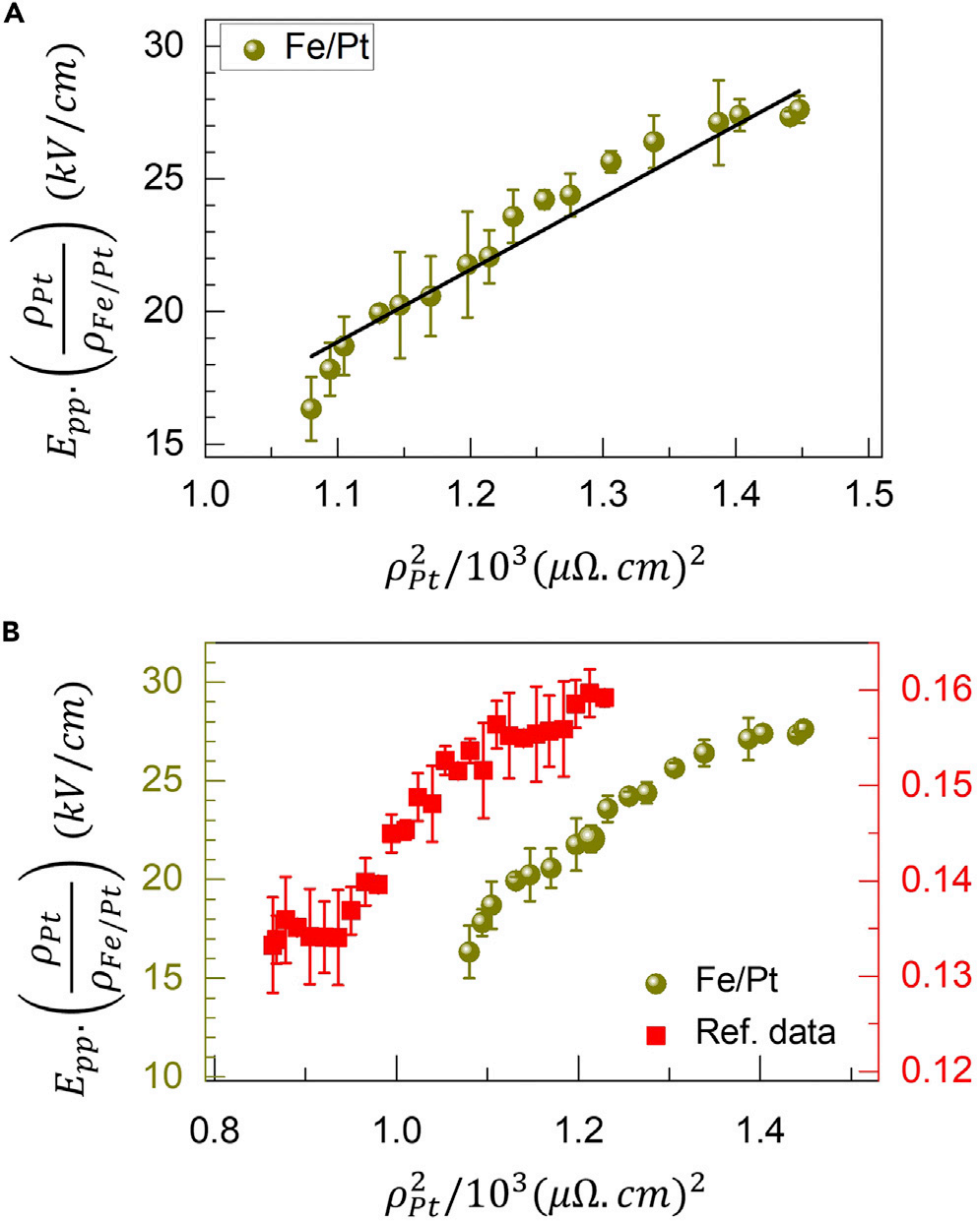
Spin Hall resistivity $\rho_{\mathrm{SH}}$ extracted from the experimentally measured THz amplitude, $E_{\mathrm{PP}}$ as a function of squared electrical resistivities $\rho^{2}$ for the Fe/Pt bilayer sample (A) The spin Hall resistivity of Pt, $\rho_{SH}^{Pt}\propto E_{PP}.\left(\frac{\rho_{Pt}}{\rho_{Fe/Pt}}\right)$ from the experimentally measured THz amplitude with respect to the electrical resistivities $\rho_{Pt}^{2}$ for the Fe/Pt bilayer. The solid black line is a linear fit to the experimental data using Equation (4). The values of slope and intercept are 27.2 and −11.1, respectively. (B) Comparison between the temperature-dependent behaviors of the data for Fe/Pt and Co/Pt. The latter (Ref. data) is taken from ref. Matthiesen et al. (2020) to confirm dominating intrinsic spin-charge conversion mechanism in the Pt-based FM/NM bilayer spintronic THz emitters. The error bars represent the maximum experimental error in the measure THz signal.

**Figure 4:**
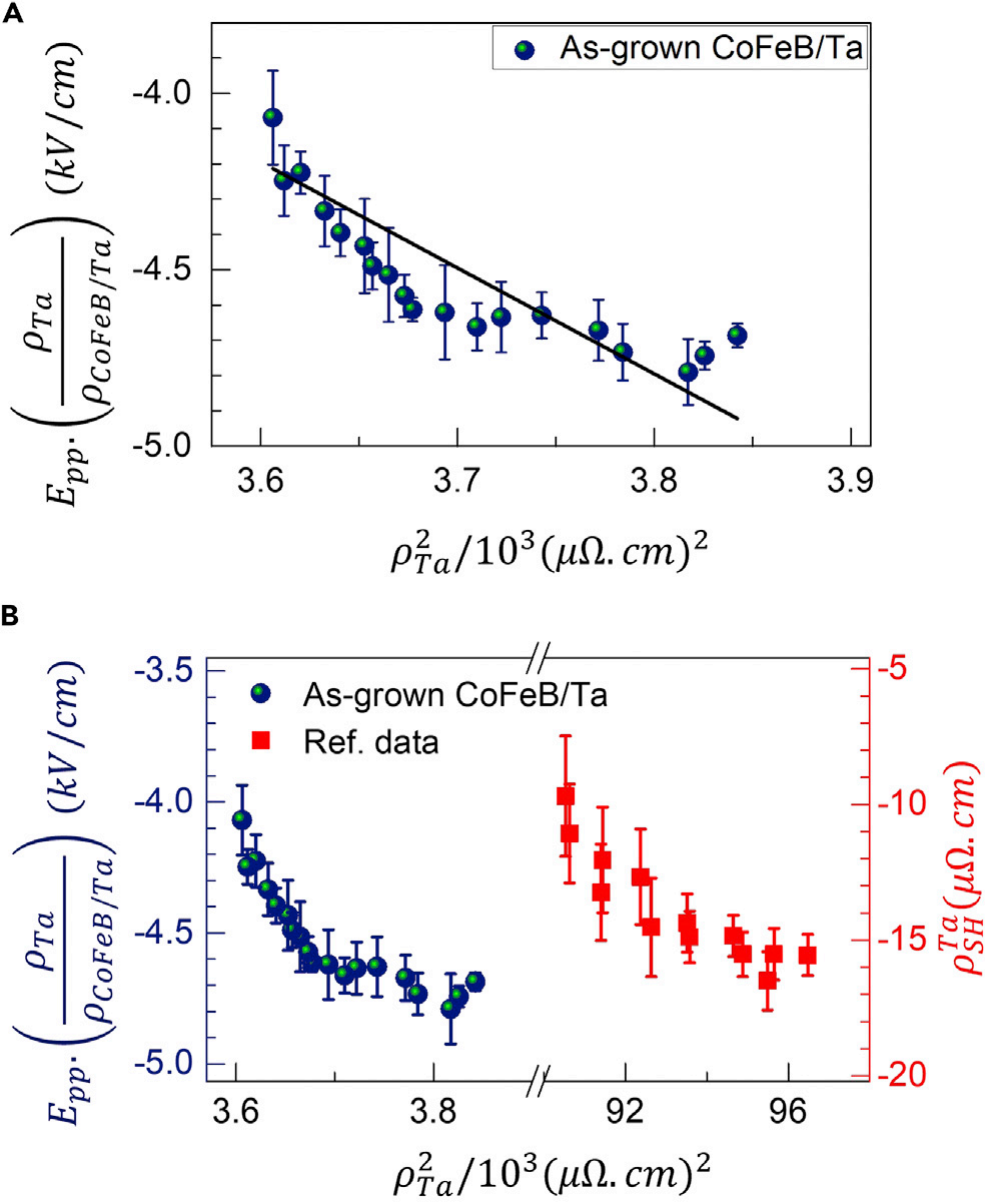
Spin Hall resistivity, $\rho_{SH}^{Ta}\;\propto E_{PP}.\left(\frac{\rho_{Ta}}{\rho_{CoFeB/Ta}}\right)$ of the α-phase Ta in the as-grown CoFeB/Ta spintronic THz emitter (A) $E_{PP}.\left(\frac{\rho_{Ta}}{\rho_{CoFeB/Ta}}\right)$ as a function of $\rho_{Ta}^{2}$ . The solid black line represents the fit to the data using Equation 4. The values of the slope and the intercept are −3 and 6.6, respectively. (B) Comparison between the spin Hall resistivity of α-phase Ta from the current study and that from ref. data Sagasta et al. (2018). The error bars represent the maximum experimental error in the measure THz signal.

**Figure 5:**
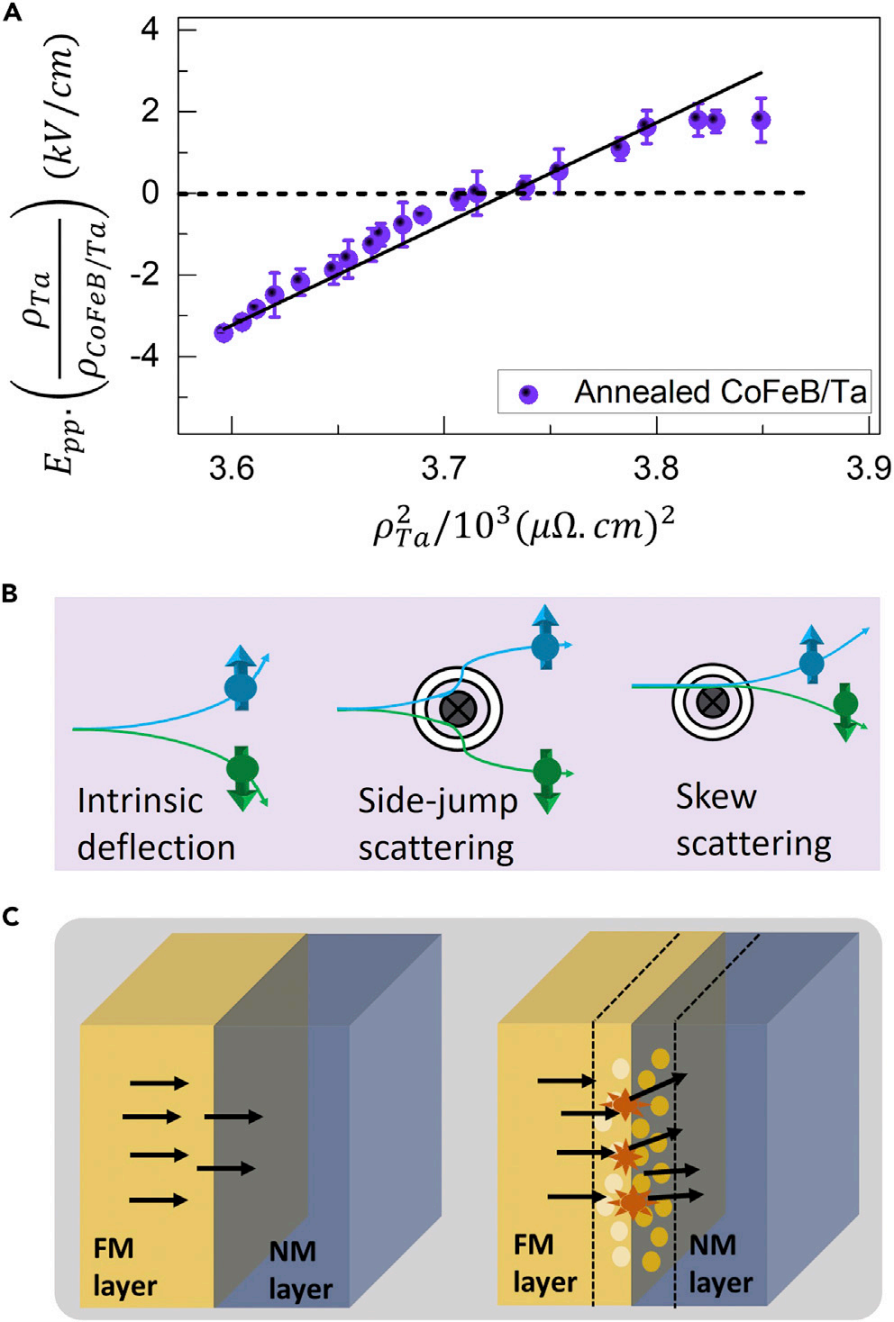
Interface modifications and effect on the spin Hall conductivity owing to the annealing of CoFeB/Ta heterostructure (A) $E_{PP}.\left(\frac{\rho_{Ta}}{\rho_{CoFeB/Ta}}\right)$ as a function of $\rho_{Ta}^{2}$ in the annealed CoFeB/Ta bilayer structure. Black solid line represents a linear fit to the data using Equation 4. The values of the slope and intercept are 24.9 and −92.3, respectively. The error bars represent the maximum experimental error in the measure THz signal. (B) Schematic deflection of the opposite spins in a heavy metal contributing to the spin-charge conversion. The deflection takes place owing to, either the intrinsic spin-orbit coupling mechanism or by extrinsic mechanisms relating to side-jump and skew scattering from impurities/defects. For a given material, both the intrinsic and the extrinsic mechanisms can contribute to the overall spin-charge conversion. (C) Schematic illustration of the annealing effect in the CoFeB/Ta bilayer in which, B diffusion across the interface can alter the interfacial properties and hence the THz emission significantly.

From the experimentally known values, $E_{PP}.\left(\frac{\rho_{HM}}{\rho_{FM/HM}}\right)$ vs $\rho_{HM}^{2}$ results are presented in Figures 3, 4, and 5 for the three bilayer heterostructures, Fe/Pt, as-grown CoFeB/Ta, and annealed CoFeB/Ta, respectively. In each case, a linear fit using Equation (4) has been used to estimate various intrinsic and extrinsic scattering parameters as desired. The slope of the linear fit provides, $\sigma_{HM}^{int}$, while the intercept provides the value of $\sigma_{SJ}.\rho_{0,HM}^{2}+\alpha_{ss}.\rho_{0,HM}$. It can be seen from Figures 3A, 4A, and 5A that the linear fits using Equation (4) are quite reasonable for all three bilayer samples, and hence the intrinsic contribution is confirmed. However, different values of the intercepts amount for the difference in the extrinsic contributions in each one of them for the spin-to-charge conversion mechanism, which is now discussed in detail in the following paragraphs.

The temperature-dependent behavior of the spin Hall resistivity, $\rho_{SH}^{Pt}$ for Pt that is proportional to $E_{PP}.\left(\frac{\rho_{Pt}}{\rho_{Fe/Pt}}\right)$ for the Fe/Pt, has been presented in Figure 3A, where the solid line is fit to the $E_{PP}.\left(\frac{\rho_{Pt}}{\rho_{Fe/Pt}}\right)$ vs $\rho_{Pt}^{2}$ using Equation (4). From the fitting, we estimate the value of the intrinsic spin Hall conductivity of Pt to be $\sigma_{Pt}^{int}∼\left(\frac{\hbar }{\text{e}}\right)413\;{\text{Ω}}^{-1}{\text{cm}}^{-1}$. Please see the STAR methods section for the details of the calculations, where a constant value of the spin current (Kampfrath et al., 2013), was used. The value of the spin Hall conductivity of Pt estimated in the current study is slightly smaller than the range reported in the literature (Isasa et al., 2015; Sagasta et al., 2016). It may be noted that for the determination of the exact value of the intrinsic spin Hall conductivity, one requires a predetermined value of the spin current density, J_s_ also, which itself can weakly depend on the temperature (Isasa et al., 2015). However, the linearly increasing behavior of the spin Hall resistivity of Pt in Fe/Pt resembles that of the other Pt-based bilayer FM/NM structures and hence the dominant role of the intrinsic spin-charge conversion mechanism in them is confirmed (Matthiesen et al., 2020). For comparison, a result for Co/Pt from the literature (Matthiesen et al., 2020) is plotted in Figure 3B, and the resemblance between the two can be clearly seen. A small and constant horizontal offset in the entire range between the data for the two Pt-based FM/NM bilayers, i.e., Fe/Pt (current study) and Co/Pt from ref. Matthiesen et al. (2020) arises from the difference in the material combinations and their resistivities. The intrinsic origin of the spin-charge conversion in Pt, as confirmed by our study, is also consistent with the other theoretical (Wang et al., 2016) and experimental (Sagasta et al., 2016; Nguyen et al., 2016) studies on similar Pt-based FM/NM bilayers.

Except for the THz signal polarity reversal, the behavior of the $E_{PP}.\left(\frac{\rho_{Ta}}{\rho_{CoFeB/Ta}}\right)$ vs $\rho_{Ta}^{2}$ data for the as-grown CoFeB/Ta, as shown in Figure 4A, appears to be similar to that for Fe/Pt. Like before, a linear fit as shown by a solid line in Figure 4A using Equation (4), provides the experimental value of the intrinsic spin Hall conductivity for the α-phase Ta layer, which comes out to be $\sigma_{Ta}^{int}∼\left(\frac{\hbar }{\text{e}}\right)\left(-46\right)\;{\text{Ω}}^{-1}{\text{cm}}^{-1}$. This value is in close consistency with the literature (Tanaka et al., 2008; Qu et al., 2018), where the spin Hall conductivity for α-phase Ta has been reported to be in the range of ∼ ‒(50–250) Ω^−1^cm^−1^. It is noteworthy to note that the value for the β-phase Ta (Sagasta et al., 2018) is relatively much higher (∼−800 Ω^−1^cm^−1^), i.e., nearly one order of magnitude higher than the α-phase Ta. Obviously, this difference in the $\sigma_{Ta}^{int.}\;$ values is owing to the difference between the resistivities in the two phases of Ta. In Figure 4B, we have re-plotted the $E_{PP}.\left(\frac{\rho_{Ta}}{\rho_{CoFeB/Ta}}\right)$ vs $\rho_{Ta}^{2}$ data for the α-phase Ta (current study) to compare it with $\rho_{SH}^{Ta}$ vs $\rho_{Ta}^{2}$ for the β-phase Ta, the latter taken from ref. Sagasta et al. (2018). Like before, a constant horizontal shift between the data for the CoFeB/Ta(α) and CoFeB/Ta(β) is because of the difference in the resistivities of the Ta layers in the two phases, particularly, the one-order smaller resistivity of Ta(α) than Ta(β). Nevertheless, nearly one-to-one similarity in the trends of the two plots is quite evident in Figure 4B, thereby confirming the predominance of the intrinsic origin for the spin-to-charge conversion mechanism in the as-grown CoFeB/Ta system.

In the case of the annealed CoFeB/Ta sample, the variation of the spin Hall resistivity, $\rho_{SH}^{Ta}\;\propto E_{PP}.\left(\frac{\rho_{Ta}}{\rho_{CoFeB/Ta}}\right)$, as shown in Figure 5A, still follows a linear relation with $\rho_{Ta}^{2}$. The zero-line crossing of the graph arises from the THz polarity reversal below a certain temperature as shown in Figure 2B. From the slope of the linear fit, the value of the intrinsic spin Hall conductivity, $\sigma_{Ta}^{int}∼\left(\frac{\hbar }{\text{e}}\right)\left(377\right)\;{\text{Ω}}^{-1}{\text{cm}}^{-1}$ is obtained. However, the intercept of the linear fit on the $\rho_{Ta}^{2}$-axis is much different, larger by at least one order than that for the Fe/Pt as well as the as-grown CoFeB/Ta. As the intercept of the linear fit is a measure of $\sigma_{SJ}.\rho_{0,HM}^{2}+\alpha_{ss}.\rho_{0,HM}$ through Equation (4), therefore, the results suggest that the extrinsic contributions to the overall spin-charge conversion in the annealed CoFeB/Ta are quite significant. Both the extrinsic and intrinsic SOC mechanisms are schematically shown in Figure 5B for duly reference. The extrinsic effect includes the side-jump and skew scattering mechanisms, which are related to the scattering events from impurity/defect centers in the material or its interface with another material (Berger, 1970; Smit, 1958).

**Figure 6:**
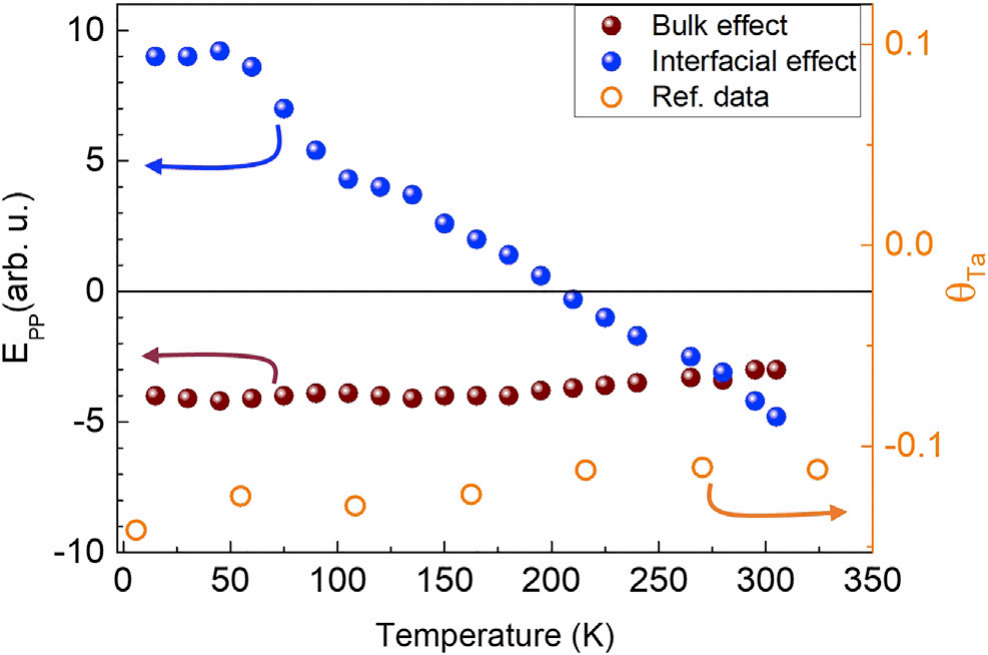
Temperature-dependent THz emission from the bulk-like intrinsic and interfacial extrinsic contributions to the overall SOC in the annealed CoFeB/Ta bilayer heterostructure The spin Hall angle data (Ref. data) plotted with open circles is taken from ref. Hao and Xiao (2015) and is shown for a direct comparison.

In the inverse SHE for the THz emission through the usual relation, $\vec{J_{c}}=\;\theta .\;\left(\;\vec{J_{s}}\times \hat{m}\right)$ , a sign change in the THz polarity below a certain temperature, in the case of annealed CoFeB/Ta as shown in Figure 2B, implies that the spin Hall angle or the spin Hall resistivity, $\rho_{SH}^{Ta}={\text{θ}}^{Ta}.{\text{ρ}}_{Ta}$ gets sign reversed. The THz charge current can be assumed to be consisting of two contributions in the spin Hall angle, one from the bulk-like intrinsic SOC and another from the interface (Hou et al., 2012). This is possible in the case of the annealed CoFeB/Ta, in which, the B-ion diffusion during the annealing process can alter the interface as compared to that in the case of the as-grown CoFeB/Ta. This possibility is schematically shown in Figure 5C that there is a perfectly sharp interface in one case and an imperfect interface in the other. The transmission of the arrows through the interface here represents the spin current conversion efficiency in the two cases. The bulk-like component of the spin Hall angle in the annealed CoFeB/Ta can be assumed to be the same as that in the as-grown CoFeB/Ta. Using the temperature-dependent values of E_PP_ in Figures 2A and 2B for the as-grown CoFeB/Ta and annealed CoFeB/Ta, respectively, both the bulk-like and the interfacial components to the E_PP_ in the annealed CoFeB/Ta are shown separately in Figure 6. Furthermore, in the same figure, the experimentally measured values of the spin Hall angle in Ta, taken from ref. Hao and Xiao (2015), have also been plotted for comparison. A close match between the spin Hall angle and the bulk-like component of E_PP_ in their temperature-dependent behavior, justifies the initial postulate of having the bulk-like intrinsic and interfacial extrinsic components of the overall spin Hall angle/SOC in the annealed CoFeB/Ta. A giant interfacial contribution to the spin Hall angle and hence the THz amplitude E_PP_ along with its sign reversal at ∼200 K in the annealed CoFeB/Ta, is clearly seen in Figure 6. A possible reason and further understanding for the observed behavior is elaborated in the discussion part.

## Discussion

In the process of THz emission from spintronic heterostructures, both the bulk-like or the intrinsic and the interfacial or the extrinsic contributions to the SOC must be considered (Zhou et al., 2018). Generally, the ISHE is at play maximally in bulk and weakly or reasonably large, depending on the conditions at the interface between two metal layers, whereas, the IREE is majorly present in the latter case only (Zhou et al., 2018; Jungfleisch et al., 2018). The dominance of one over the other depends on the nature of the interface. For example, in an FM/NM heterostructure using Ag/Bi layer combination for the NM, the Ag/Bi interface was well understood as a Rashba interface in which the SOC was mainly governed by the IREE (Jungfleisch et al., 2018). However, if Ag_x_Bi_100-x_ alloy is used as the NM layer, it was found that the interfacial contribution from ISHE can be significantly larger than the IREE for certain compositions (Shen et al., 2021). A dominant contribution from an interface-related extrinsic mechanism over the intrinsic one for the spin-charge conversion has also been indicated in a few previous studies on other material structures. In a theoretical study (Wang et al., 2016), it was shown that the value of the spin Hall angle in pure bulk Pt can get enhanced by more than an order owing to the additional contribution from its interface with an FM layer of permalloy (Py). Bismuth (Bi) has a relatively much smaller value of the intrinsic spin Hall angle than Pt; however, in a Py/Bi heterostructure, the overall spin Hall angle that is majorly contributed by the interfacial extrinsic mechanism becomes much higher than the intrinsic one in Pt alone (Hou et al., 2012).

To probe the interface-related phenomena, THz emission spectroscopy is realized to be as effective as the steady-state FMR-based techniques because of the interrelated total SOC and spin-orbit torque mechanisms. It is known that in presence of an interlayer, the magnitude of THz emission through ISHE in an FM/NM spintronic heterostructure follows the same trend as that of the Gilbert damping parameter with respect to the thickness of the interlayer (Hawecker et al., 2021). The latter was measured by using steady-state FMR technique. The spin-orbit torque has two components, if disentangled, the damping-like (DL) and the field-like (FL). There can be only DL torque from the intrinsic bulk part of the SHE, while from the interface, among the DL and the FL torques, the latter dominates as seen earlier from spin-torque FMR and harmonic Hall measurements (Du et al., 2020; Pai et al., 2015; Allen et al., 2015). CoFeB/Ta is a system, where harmonic Hall measurements have revealed significant contribution from the FL torque along with the intrinsic DL contribution (Allen et al., 2015). Therefore, the present results are indicative that the FL torque contributes differently in the as-grown and annealed CoFeB/Ta bilayers. The annealing process causes boron deficiency in CoFeB and hence the composition around the interface and its nature gets altered. The interfacial modifications in the annealed CoFeB/Ta are evident from the XRD and MH measurements also. Please refer to Figures S1 and S2 in the supplemental information section. The sign reversal in the THz polarity at ∼200 K arises from the interfacial effect for the extrinsic contribution to the total SOC in the annealed CoFeB/Ta (Figure 6). This particular result would analogously correspond to the sign reversal of interfacial FL torque (Du et al., 2020; Pai et al., 2015) in temperature-dependent spin-torque FMR and harmonic Hall measurements.

### Conclusion

In conclusion, we have studied the spin-charge conversion process through THz emission measurements on the as-grown and annealed CoFeB/Ta bilayers spintronic THz emitters and compared them with an extensively studied Fe/Pt system. Detailed temperature-dependent measurements have helped determine the behavior of the spin Hall resistivity and the contributions from the intrinsic and extrinsic components in it. The linear proportionality of the spin Hall resistivity with the squared resistivity of Pt and α-phase Ta in Fe/Pt and the as-grown CoFeB/Ta, respectively, indicate the dominance of intrinsic bulk contribution to the overall spin-charge conversion mechanism in them. It has been found that the THz pulse generated from the annealed CoFeB/Ta gets polarity reversed below a certain temperature, which is solely related to the sign reversal in the interfacial spin Hall angle. This peculiar behavior of the sign reversal of the spin Hall angle at ∼200 K clearly suggests a significant role of the interfacial contributions along with the intrinsic contribution to the inverse SHE, which to our best knowledge, has not been seen hitherto. In addition, our measurements also strengthen the point further that ultrafast THz emission can be used for studying the microscopic SOC in the spintronic structures in a contactless and non-destructive manner through the time-domain THz spectroscopy.

### Limitations of the study

The experimental determination of the field-like and damping-like spin-orbit torque components and their dependency on the temperature can also be conducted using complimentary FMR measurements that will help provide further insights. More low-temperature ultrafast THz emission investigations on other possible FM/NM combinations such as CoFeB/Pt, Fe/Ta, and so forth and the effect of annealing can be carried out in future to control and quantify the role of the interfaces better.

## STAR★Methods

### Key resources table

**Table:** 

REAGENT or RESOURCE	SOURCE	IDENTIFIER
**Chemicals, peptides, and recombinant proteins**		

Iron (Fe) material sputtering Target	ACI Alloys	CAS# 7439-89-6
Platinum (Pt) material sputtering Target	ACI Alloys	CAS# 7440-06-4
Tantalum (Ta) material sputtering Target	ACI Alloys	CAS# 7440-25-7
Co_20_Fe_60_B_20_ (Cobalt Iron Boron) material sputtering Target	ACI Alloys	NA

**Software and algorithms**		

Origin 8	Origin Lab	https://www.originlab.com/

### Resource availability

#### Lead contact

Further information and requests for resources should be directed to and will be fulfilled by the lead contact, Prof. Sunil Kumar (kumarsunil@physics.iitd.ac.in).

#### Material availability

This study did not generate new unique reagents.

### Experimental model and subject details

This study does not use experimental methods typical in the life sciences.

### Method details

#### Sample preparation and characterization

High-quality thin-film FM/NM bilayer heterostructures of the ferromagnetic and nonmagnetic materials were fabricated by ultra-high vacuum RF/DC magnetron sputtering, and they were used as spintronic THz emitters in a time-domain spectrometer for the results presented here. The thin film deposition order was substrate/FM/NM, and no *in situ* substrate heating protocol was used. The CoFeB(3nm)/Ta(3nm) and Fe(3nm)/Pt(3nm) bilayers were deposited on pre-treated high-resistive <100 > silicon substrates (HR-Si) having thickness Si(380 mm)/SiO_2_(0.1 mm) in an ultra-high vacuum with a base pressure value lower than 6 × 10^−8^ Torr. Under pre-treatment procedure, the substrates were chemically cleaned by double ultrasonication in acetone and isopropyl alcohol for removing various types of impurities from the silicon surface. For the CoFeB/Ta and Fe/Pt bilayers, the thicknesses of FM and NM layers are such that a reasonably high THz signal is obtained from those combinations (Kumar et al., 2021b). A separate set of samples having individual layers of Pt (3nm) and Ta (3nm) were also deposited. Another piece of the CoFeB/Ta heterostructure was post-annealed for 1 h at a temperature of 350°C under the same base pressure value as mentioned above. The crystalline phase, thickness, roughness, etc., were obtained by the optimized growth parameters during the deposition process and also reconfirmed through X-ray diffraction (XRD) and X-ray reflectivity (XRR) measurements (Kumar et al., 2021b). The X-ray diffraction (XRD) measurements were carried out using a PANalytical X’Pert diffractometer with a Cu-K_α_ source on CoFeB/Ta thin films. Figure S1 shows the GIXRD (grazing incident angle X-ray diffraction) patterns of the as-grown and annealed CoFeB/Ta heterostructure sample grown on the silicon substrate. The diffraction peaks observed in Figure S1 at angles of ∼38° and ∼55° in the case of an as-grown sample are due to the α-phase Ta and the substrate. The absence of any other peak indicates the amorphous phase of CoFeB in the as-grown CoFeB/Ta heterostructure. For the annealed CoFeB/Ta sample, an additional Bragg peak at ∼45° angle is observed, which is related to the bcc crystal structure of CoFe, evidently suggesting that boron diffusion at the interface with the adjacent Ta layer has taken place (Bouchikhaoui et al., 2013). The above fact is consistently seen in the magnetic hysteresis (M−H) measurements performed on both the as-grown and annealed CoFeB/Ta samples. The substrate-corrected hysteresis loops are shown in Figure S2. The hysteresis curve of the annealed sample shows a larger value of both the coercive field and the saturation magnetization than the as-grown CoFeB/Ta sample. This observation is attributed to the recrystallization of the CoFeB layer in the annealed CoFeB/Ta heterostructure due to the process of annealing at ∼625 K in our experiments. The observed MH-behaviour of the as-grown and the annealed CoFeB/Ta samples is consistent with the literature (Burrowes et al., 2013; Swamy et al., 2013).

#### THz time-domain spectroscopy

For all the temperature-dependent electrical transport and THz time-domain emission measurements, we have used a closed-cycle helium cryostat system operating in the temperature range of ∼5–450 K. The temperature-dependent resistance (R-T) measurements were performed using four-point van der Pauw method. The layout of the home-built THz time-domain spectroscopy setup with the low-temperature cryostat integrated into it, is shown in Figure 1A. A femtosecond (fs) laser beam of 800 nm central wavelength, 1 kHz pulse repetition rate, and ∼50 fs pulse duration is divided into two parts using a 90:10 beam splitter. The strong part is used to optically excite the spintronic emitter mounted on the cold finger of the cryostat chamber. The collimated excitation beam diameter was ∼3 mm, while the fluence at the sample point was fixed at ∼1 mJ/cm^2^. At this fluence value, the optical pump induced changes in the reflection and transmission properties of the substrate are nearly unaffected (Degert et al., 2022). The emitted THz radiation is then collected by gold-coated 90° off-axis parabolic mirrors and focused onto a 500 mm thick (110)-oriented ZnTe crystal for detection. A high-resistive silicon wafer is placed just after the sample in order to separate the emitted THz radiation from the residual optical beam. The weak portion from the fs laser beam was used as gating beam for the THz detection. These time-delayed (t) gating pulses are made pass through a hole in the last parabolic mirror in the setup to coincide temporally and spatially with the collinearly propagating THz beam onto the ZnTe crystal (Figure 1A) for the detection of the latter by electro-optic sampling scheme. More details about the experimental setup can be found elsewhere (Kumar et al., 2021c, 2021d). The spintronic emitters were magnetized above the saturation magnetic field (Kumar et al., 2021a, 2021b) of the device using an *in situ* external magnetic field (∼200 milli-Tesla) equipped within the cryostat chamber (Figure 1A).

#### Estimation of the strength of the emitted THz pulse electric field

The THz electric field emitted from the spintronic emitters in the experiments can be reconstructed from the measured electro-optic signal sampled on a ZnTe crystal by following the standard method(Planken et al., 2001; Cheng et al., 2019; Yang et al., 2016). According to this, the THz electric field can be expressed in terms of the differential intensity measured on the balanced photodiode as following

(Equation 5) $$E_{\mathrm{THz}}\left(\frac{V}{\mathrm{cm}}\right)=\left(\frac{\Delta I}{I}\right)\frac{2c}{\omega n^{3}r_{41}L}\left(cos\alpha \;sin2\beta +2sin\alpha \;cos2\beta \right)$$

Here, c = 3 × 10^8^ m/s is the speed of light in vacuum, ω = 2πν = 2πc/800nm is the angular frequency of the gating beam as shown in Figure 1 of the manuscript. The thickness of the ZnTe crystal was L = 0.5mm. The optical constants of the crystal are: refractive index (Li, 1984), n = 2.85 and nonlinear coefficient (Planken et al., 2001), r_41_ = 3.9 × 10^−12^ m/V α and β are the angles of THz beam polarization and gating beam polarization with respect to the c-axis (001) of the ZnTe crystal, which are kept at 90° and 180°, respectively, in our experiment. With all these values, Equation 5 reduces to $\;E^{THz}=28.2\left(\frac{\Delta I}{I}\right)\left(\frac{kV}{cm}\right)$. The quantity ΔI/I represents the differential change in the gating beam intensity on the balanced photo detector.

#### Intrinsic Spin-hall conductivity from the emitted THz electric field strength

According to Equation 2, the spin Hall resistivity in terms of THz electric field can be written as

(Equation 6) $$\rho_{\mathrm{SH}}=E_{\mathrm{THz}}{\left(\frac{1}{d}\cdot \frac{\rho_{\mathrm{FM}/\mathrm{NM}}}{\rho_{\mathrm{NM}}}\right)}^{-1}.\;\frac{1}{J_{s}}$$

Also, the spin Hall resistivity has a linear relationship with the squared longitudinal resistivity, $\left(\rho_{\mathrm{NM}}^{2}\right)$, whose slope is equal to the intrinsic spin Hall conductivity of the NM layer (see Equation 3). Hence, intrinsic spin Hall conductivity can be rewritten in the form of the THz electric field as follows

(Equation 7) $$\sigma_{\mathrm{NM}}^{\mathrm{int}.}.\rho_{\mathrm{NM}}^{2}=E_{\mathrm{THz}}{\left(\frac{1}{d}\cdot \frac{\rho_{\mathrm{FM}/\mathrm{NM}}}{\rho_{\mathrm{NM}}}\right)}^{-1}.\;\frac{1}{J_{s}}\;$$

(Equation 8) $$\text{or}\;\sigma_{\mathrm{NM}}^{\mathrm{int}.}=\;\frac{E_{\mathrm{THz}}{\left(\frac{1}{d}\frac{\rho_{\mathrm{FM}/\mathrm{NM}}}{\rho_{\mathrm{NM}}}\right)}^{-1}}{\rho_{\mathrm{NM}}^{2}}.\;\frac{1}{J_{s}}$$

where, the first term in Equation 8 is nothing but the value of the *slope* obtained from the $E_{\mathrm{THz}}{\left(\frac{1}{d}\cdot \frac{\rho_{\mathrm{FM}/\mathrm{NM}}}{\rho_{\mathrm{NM}}}\right)}^{-1}$ versus $\rho_{\mathrm{NM}}^{2}$ plot as discussed before. *J*_*s*_ is the spin current density generated from the ultrafast optical excitation of the FM/NM spintronic heterostructure. Assuming superdiffusive process, the spin current density averaged over the thickness (*J*_*s*_*/d*) in such heterostructures (Kampfrath et al., 2013) is typically ∼10^30^ A/m^2^. By considering the above, Equation 8 can be rewritten as

(Equation 9) $$\sigma_{\mathrm{NM}}^{\mathrm{int}.}=\left(\mathrm{slope}\right).\;\frac{10^{2}}{6.58}\left(\frac{\hbar }{e}\right)\left(\Omega^{-1}cm^{-1}\right)$$

Equation 9 has been used to calculate the intrinsic spin Hall conductivity for all samples under study. The exact value of the intrinsic spin Hall conductivity may differ because we have used theoretically calculated value of the spin current density, and also the underestimated temperature variation of spin relaxation length and spin current density.

## Data Availability

Data reported in this paper will be shared by the lead contact upon request. There is no dataset or code associated with this work. Temperature-dependent THz emission, Spintronic heterostructures, Annealed CoFeB/Ta, interfacial spin-charge conversion, Inverse spin Hall effect.
