# Supplementary material for: Large interfacial contribution to ultrafast THz emission by inverse spin Hall effect in CoFeB/Ta heterostructure
Source: iScience. 2022 Jul 4;25(8):104718. doi: 10.1016/j.isci.2022.104718 (PMC9293784; doi:10.1016/j.isci.2022.104718)
Supplement: Document S1. Figures S1 and S2 [file mmc1.pdf]

iScience, Volume *25*

Supplemental information

Large interfacial contribution to ultrafast THz emission by inverse spin Hall effect in CoFeB/Ta heterostructure

Sandeep Kumar and Sunil Kumar

Supplementary Information

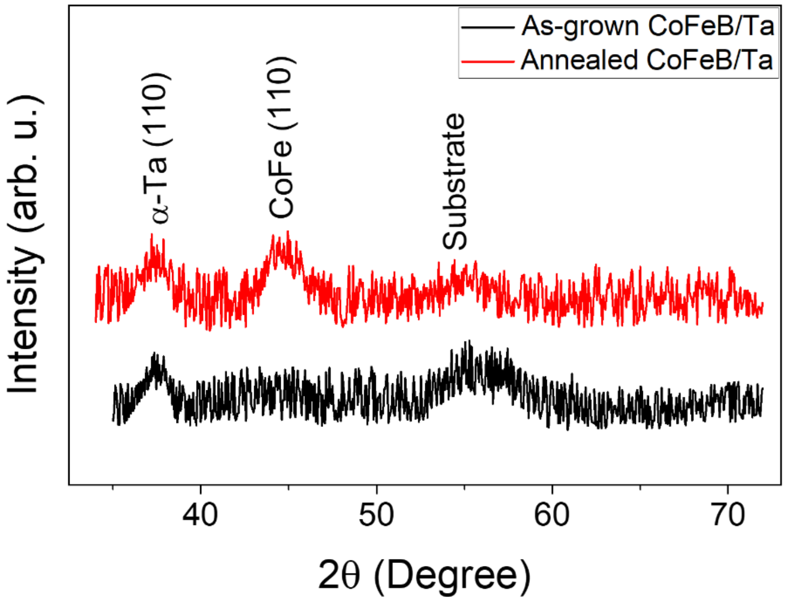

Figure S1. XRD plots of as-grown and annealed CoFeB/Ta spintronics heterostructures. The peaks and corresponding crystalline planes are indicated. Related to **Star Method**.

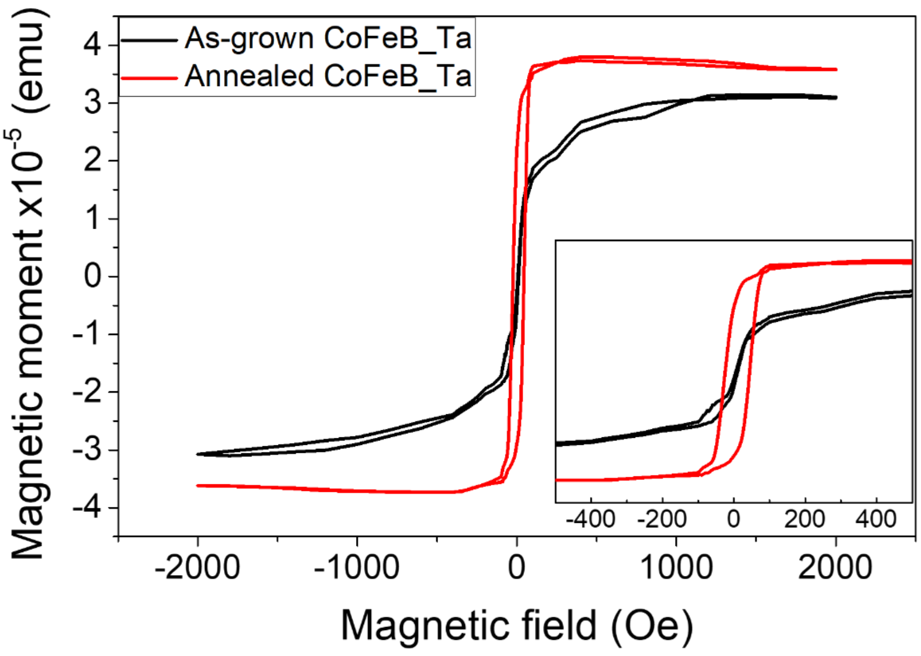

Figure S2. In-plane hysteresis loops from MH measurement on the as-grown and annealed CoFeB/Ta heterostructures. Inset: zoomed-in view for clarity. Related to **Star Method**.
